# Localization of Flavan-3-ol Species in Peanut Testa by Mass Spectrometry Imaging

**DOI:** 10.3390/molecules25102373

**Published:** 2020-05-20

**Authors:** Hirofumi Enomoto, Takashi Nirasawa

**Affiliations:** 1 Department of Biosciences, Faculty of Science and Engineering, Teikyo University, Utsunomiya 320-8551, Japan; 2 Division of Integrated Science and Engineering, Graduate School of Science and Engineering, Teikyo University, Utsunomiya 320-8551, Japan; 3 Advanced Instrumental Analysis Center, Teikyo University, Utsunomiya 320-8551, Japan; 4 Application Department Daltonics Division, Bruker Japan K.K., Yokohama 221-0022, Japan; takashi.nirasawa@bruker.com

**Keywords:** peanut testa, procyanidins, flavan-3-ols, mass spectrometry imaging (MSI), matrix assisted laser desorption/ionization (MALDI), matrix vapor deposition/recrystallization

## Abstract

Flavan-3-ols, procyanidins and their monomers are major flavonoids present in peanuts that show a wide range of biological properties and health benefits, based on their potent antioxidant activity. Procyanidin oligomers, especially A-type, are reportedly abundant in peanut skin; however, their localization in the raw peanut testa remains poorly understood. Therefore, we performed matrix-assisted laser desorption/ionization-mass spectrometry imaging (MALDI-MSI) to investigate the localization of flavan-3-ols in peanut testa. 1,5-Diaminonaphthalene was coated onto the peanut section by matrix vapor deposition/recrystallization, and MALDI-MSI measurements were performed in the negative-ion mode. Peaks matching the *m*/*z* values of flavan-3-ol [M − H]^−^ ions were observed in the mass spectrum extracted from the outer epidermis of the peanut testa, using the region of interest function. Catechin and/or epicatechin, five A-type, and one B-type procyanidins were assigned by the fragment ions generated by retro-Diels-Alder, heterocyclic ring fission, and quinone methide reactions detected in MALDI-tandem MS spectra. These flavan-3-ols were localized in the outer epidermis of the peanut testa. This information will contribute to improving the extraction and purification efficiencies of flavan-3-ols from peanut testa. As flavan-3-ols display anti-microbial activity, it is speculated that flavan-3-ols present in the outer epidermis of peanut testa act to prevent pathogen infection.

## 1. Introduction

**Figure 1:**
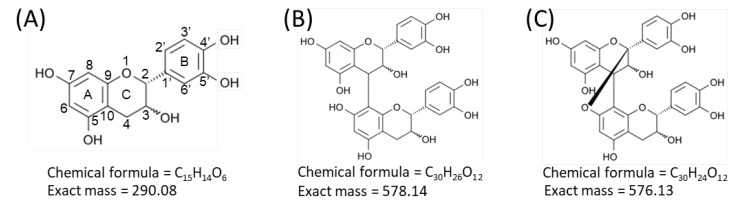
Structures of flavan-3-ol species. Structures of (**A**) catechin and epicatechin, flavan-3-ol units of procyanidin species, (**B**) B-type procyanidin dimers, and (**C**) A-type procyanidin dimers.

Proanthocyanidins, also known as condensed tannins, are oligomers and polymers of flavan-3-ol units [1,2]. Flavan-3-ols are one of the most ubiquitous groups of plant phenolics, and are widely distributed in plants [1,2]. Flavan-3-ols are secondary metabolites biosynthesized by plants for diverse biological activities, including protection against ultraviolet radiation and pathogen attack (both bacterial and fungal) [1,2]. Flavan-3-ols are also widely distributed in human food of plant origin, particularly fruits, cereal grains, and legume seeds, in which they contribute to the bitter flavor and astringency [1,2]. In recent years, considerable attention has been paid to proanthocyanidins and their monomers, owing to their potential benefits to human health, due to their potent antioxidant properties [1,2,3,4,5]. Procyanidins, a subclass of proanthocyanidins, comprise catechin and/or epicatechin constituent units (Figure 1A) [1,2]. In the B-type procyanidins, the flavan-3-ol units are connected through a single bond between C-4 of the upper unit and C-6 or C-8 of the lower unit (Figure 1B) [1,2]. The B-type procyanidins dominate in dietary important sources such as apples and cocoa-containing foods [2,3]. A-type procyanidins differ from the B-type by having an additional bond between adjacent flavan-3-ol units that connects C-2 of the upper unit via an oxygen atom to C-7 of the lower unit (Figure 1C) [1,2].

Peanut skins are a byproduct of the peanut industry known as a rich source of procyanidin oligomers containing dimers, trimers, and tetramers, especially of the A-type [3,6]. The peanut skin extract was shown to exhibit hypocholesterolemic, anti-inflammatory, and antiallergic activities [3,4,5]. Hence, the type and content of procyanidins in the peanut skin have been investigated using modern analytical techniques, such as liquid chromatography (LC)-electrospray ionization (ESI)-mass spectrometry (MS), and/or nuclear magnetic resonance (NMR) spectroscopy [3,6]. However, their localization within the raw peanut testa has not been fully determined, because these techniques require extraction of the analyte of interest from the sample. Peanut testa contains five kind of cells containing outer epidermis, spongy parenchyma, and inner epidermis [7]. Understanding their localization in the peanut testa is considered highly desirable for facilitating their industrial utilization.

Mass spectrometry imaging (MSI) is a powerful tool to analyze the spatial distribution of diverse compounds in biological tissues [8,9,10,11,12,13,14]. In MSI, soft ionization techniques containing matrix-assisted laser desorption/ionization (MALDI) [15,16,17,18,19,20,21,22] or desorption electrospray ionization [23] have mainly been used for analysis of metabolites in plant and animal tissues. In a previous study, we used 1,5-diaminonaphthalene (DAN) as the matrix for MALDI-MSI analysis [20] to visualize flavan-3-ol species in strawberry fruit, namely properalgonidins, procyanidins and their monomers, and to analyze their distribution.

In MALDI-MSI, the fabrication of a homogeneous analyte-containing matrix coating onto the sample surface is a prerequisite for both high sensitivity and high spatial resolution [24,25]. Spraying a matrix in solution is commonly used for matrix coating; however, this involves the risk of lateral analyte delocalization. On the other hand, in recent years, matrix vapor deposition/recrystallization has received much attention for its potential to overcome these problems [24]. Thus, for example, phospholipids in different cell layers of blood vessel walls were successfully visualized with high quality, using DAN by this matrix coating method for MALDI-MSI analysis [24]. In contrast, the application of matrix vapor deposition/recrystallization for analysis of plant tissues has been limited.

In this study, we aimed to identify A-type and B-type procyanidin species using MALDI-MS/MS analysis. Subsequently, we attempted to visualize their localization within the peanut testa using matrix vapor deposition/recrystallization for MALDI-MSI analysis.

## 2. Results

### 2.1. Mass Spectra Obtained from Peanut Section

**Figure 2:**
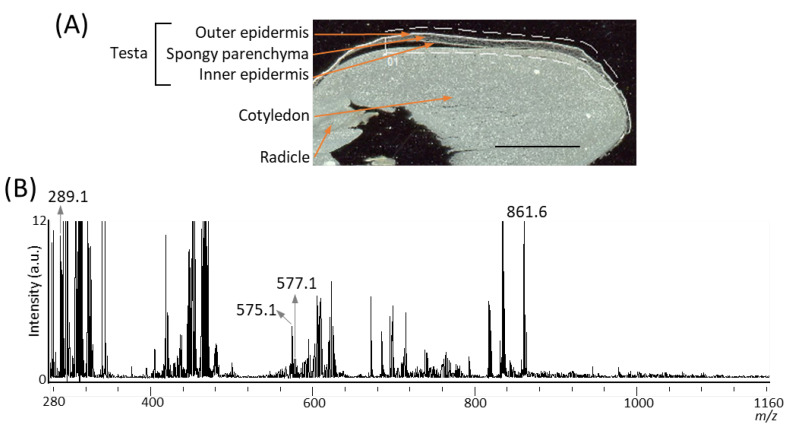
Matrix assisted laser desorption/ionization (MALDI)-mass spectrometry imaging (MSI) of a peanut longitudinal section. (**A**) Optical image of a peanut fruit section before MALDI–MSI measurement. The dotted white line shows the analyzed region. Scale bar = 5 mm. (**B**) Mass spectrum obtained from the peanut section.

It has been reported that several A-type and B-type procyanidin oligomers, and their flavan-3-ol monomers, are present in peanut skin [3,6]. In our previous study on the visualization of flavan-3-ols in strawberry fruit [20], spraying DAN in aqueous methanol was used as the matrix coating. To investigate whether procyanidins and their monomers can be detected in peanut longitudinal sections using matrix vapor deposition/recrystallization, we performed MALDI-MSI analyses. The negative-ion mode was selected over the positive-ion mode, because the procyanidin signals were concentrated into one abundant deprotonated molecule, [M − H]^−^ ion, instead of being distributed among mixtures of [M + H]^+^, [M + Na]^+^, and [M + K]^+^ adducts [2,26]. Figure 2A shows the optical image of a peanut section before MALDI-MSI. Three tissues, namely, testa, cotyledon, and radicle were observed. The region surrounded by the dotted white line was measured because, in peanut, phenolic compounds containing flavan-3-ols are predominantly present in the skin [3,6]. Figure 2B shows a mass spectrum ranging from a *m*/*z* of 280 to 1160 obtained by MALDI-MSI. In the *m*/*z* range from 1160 to 2000, peaks were hardly detected, suggesting that procyanidin pentamers and hexamers could not be detected. In the mass spectrum, peaks at *m*/*z* 289.1, corresponding to flavan-3-ol monomers, (epi)catechin, [M − H]^−^ ions, were observed. The chirality of C-3 in flavan-3-ols could not be differentiated by the MS analysis performed in this study; therefore, (epi)catechin represented either catechin or epicatechin. Peaks corresponding to *m*/*z* values of A-type and B-type procyanidin dimers [M − H]^−^ ions at *m*/*z* 575.1, and 577.1 were also observed. Although the content of procyanidin trimers and tetramers in the peanut skin were higher than those of the monomers and dimers [3], peaks corresponding to these *m*/*z* values could not be observed (Figure 2B).

### 2.2. Visualization of Flavan-3-ols in Peanut Sections

**Figure 3:**
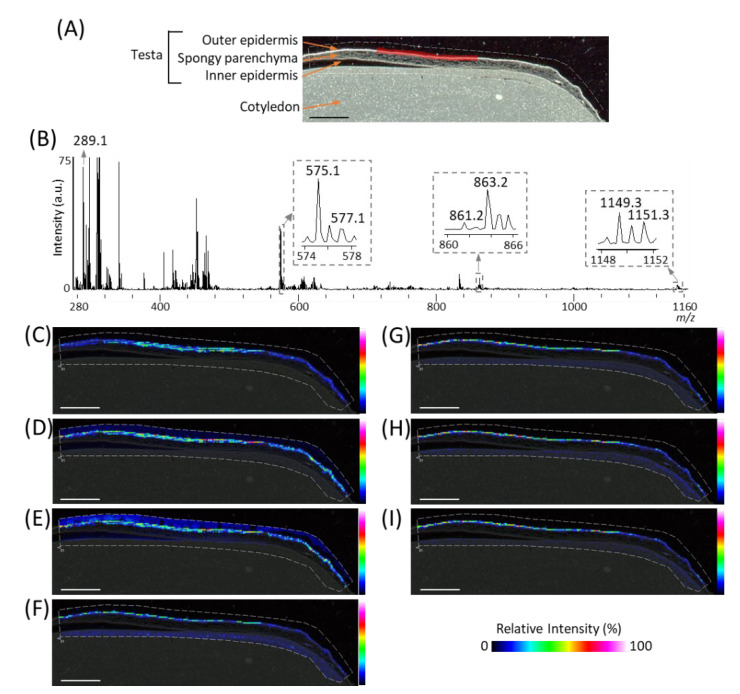
Matrix assisted laser desorption/ionization (MALDI)-mass spectrometry imaging (MSI) analysis of the outer epidermis of the peanut testa. (**A**) Optical image of the peanut longitudinal section before MALDI-MSI analysis. This is the same optimal image shown in Figure 2A. (**B**) Mass spectrum extracted from the outer epidermis of the peanut testa is indicated by the region filled in red in Figure 3A using the region of interest function. The *m*/*z* values indicated that the peaks were possibly attributable to flavan-3-ols. Representative ion images at *m*/*z* (**C**) 289.1, (**D**) 575.1, (**E**) 577.1, (**F**) 861.2, (**G**) 863.2, (**H**) 1149.3, and (**I**) 1151.3. The dotted white line shows the analyzed region. Scale bar = 2 mm.

In order to investigate the spatial distributions of *m*/*z* 289.1 (procyanidin monomers), 575.1 (A-type procyanidin dimers), and 577.1 (B-type procyanidin dimers), we reconstructed their ion images (Figure 3C–E), and found that these peaks were located in the outer epidermis of the peanut testa. In our previous study using MALDI-MSI analysis of strawberry fruit [20], procyanidin monomers, and oligomers showed similar distribution patterns because of in-source fragmentation of procyanidin species. Therefore, to look for the peaks corresponding to procyanidin trimers and tetramers, a mass spectrum was extracted from the outer epidermis of the peanut testa shown by the red region (Figure 3A), using the region of interest function (Figure 3B). The region of interest function is a function of the flexImaging software; it can extract the mean mass spectrum from specific regions. In the mass spectrum obtained, peaks at *m*/*z* 861.2 and 863.2, or 1149.3 and 1151.3, corresponding to A-type procyanidin trimers or tetramers, were observed. These ions were also found located on the outer epidermis of peanut testa (Figure 3F–I), together with procyanidin monomers and dimers (Figure 3C–E). In this study, peaks derived from phosphatidylinositol molecular species (*m*/*z* 861.6), close to the *m*/*z* values of procyanidin tetramers, were strongly detected on the cotyledon (Figure 2B). Hence, the reason peaks corresponding to procyanidin trimers could not be observed in the mass spectrum of the peanut section (Figure 2B) was the peak interference by the phosphatidylinositol molecular species. This suggests that, in the MALDI-MSI, the region where the analytes are predominantly present is preferable for mass spectral analysis. In addition to the outer epidermis of testa, ions at only *m*/*z* 861.2, 863.2, 1149.3, and 1151.3 were also observed in the cotyledon (Figure 3F,G, Supplementary Figure S1). This may be due to the contamination of other metabolites, such as phosphatidylinositol molecular species close to the *m*/*z* values of these ions, being present in the cotyledon.

### 2.3. Identification of Flavan-3-ols in Peanut Sections

**Figure 4:**
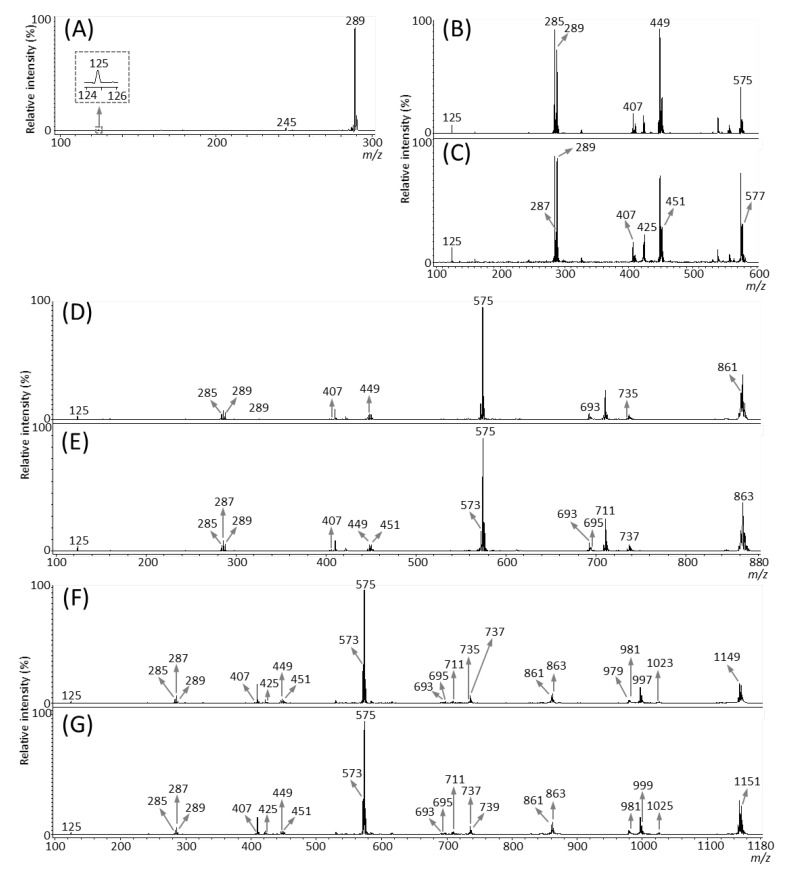
Matrix-assisted laser desorption/ionization (MALDI)-tandem mass spectrometry (MS/MS) analysis of a peanut section. MALDI-MS/MS spectra were obtained from the outer epidermis of the testa of the peanut section after MALDI-mass spectrometry imaging. Representative MS/MS spectra of precursor [M − H]^−^ ions at *m*/*z* (**A**) 289.1, (**B**) 575.1, (**C**) 577.1, (**D**) 861.2, (**E**) 863.2, (**F**) 1149.3, and (**G**) 1151.3. The fragment ion peaks with *m/z* values generated by retro-Diels-Alder (RDA), heterocyclic ring fission (HRF), and quinone methide (QM) reactions were used for molecular assignments.

We then performed MALDI-tandem MS (MS/MS) analysis to investigate whether the observed peaks at *m*/*z* 289.1, 575.1, 577.1, 861.2, 863.2, 1149.3, and 1,151.3 (Figure 3B) were attributable to flavan-3-ol monomers or oligomers [M − H]^−^ ions. The MS/MS spectra were obtained from the outer epidermis of the peanut testa section because the potential components for flavan-3-ols were localized in this tissue (Figure 3C–I). Fragment ions at *m*/*z* 245 and 125 were observed in the MS/MS spectrum of precursor ions at *m*/*z* 289.1 (Figure 4A). This fragmentation pattern agreed with the MS/MS spectra of both catechin and epicatechin standards reported previously [20]. Therefore, we assigned the peak at *m*/*z* 289.1 in peanut sections as catechin and/or epicatechin [M − H]^−^ ions.

**Figure 5:**
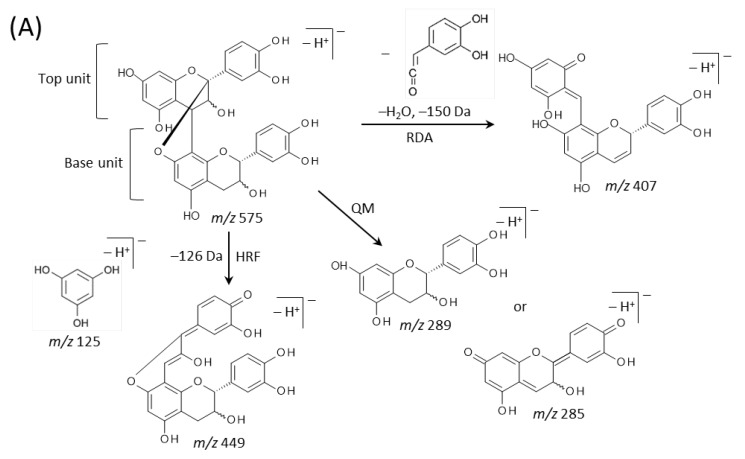

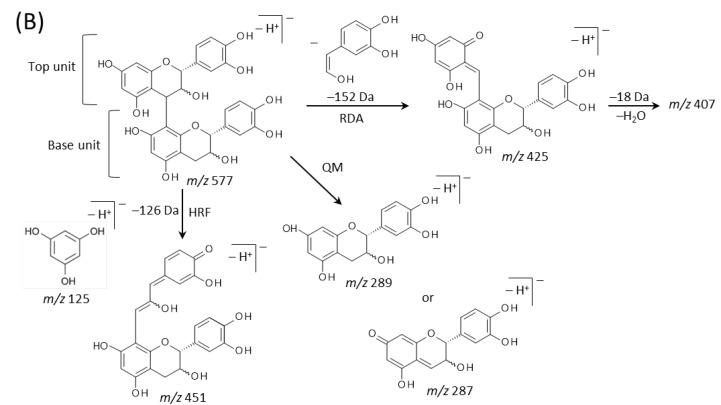
Typical fragmentation pattern of (**A**) A-type and (**B**) B-type procyanidin dimers. The characteristic fragmentation mechanisms by negative-ion mode are retro-Diels-Alder (RDA), heterocyclic ring fission (HRF), and quinone methide (QM) reactions.

Typical fragmentation patterns of A-type and B-type procyanidins observed by negative-ion mode of LC-MS/MS or MALDI-MS/MS—following retro-Diels-Alder (RDA), heterocyclic ring fission (HRF), and quinone methide (QM) reactions—were reported previously [2,26]. Figure 5 shows the typical fragmentation pattern of A-type and B-type procyanidin dimers. In the case of QM reaction, characteristic fragment ion-peaks corresponding to procyanidin monomers and oligomers with a lower degree of polymerization against precursor ions were generated for A-type and B-type procyanidins [2,26] (Figure 5A,B). In the case of RDA reaction, the fragment ions formed by the elimination of hydroxyvinyl benzenediol, [M − H − 152]^−^, and the loss an additional molecule of water, [M − H − 152 − 18]^−^ ions were characterized for B-type procyanidins (Figure 5B) [2,26], while, the single fragment ion [M − H − 168]^−^ was formed for A-type linkage of A-type procyanidin (Figure 5A) [2,26]. In the case of HRF reaction, the elimination of the 1,3,5-trihydroxybenzene, [M − H − 126]^−^ ion was characterized for A-type and B-type procyanidins, (Figure 5A,B) [2,26]. In this case, 1,3,5-trihydroxybenzene [M − H]^−^ ion was detected at *m*/*z* 125 [2,25], and the peak was clearly observed in each MS/MS spectrum obtained from the peanut sections (Figure 4B–G). In each MS/MS spectrum of precursor ions at *m*/*z* 575.1, 577.1, 861.2, 863.2, 1149.3, and 1151.3, most fragment ions with *m*/*z* values matching the expected ions following QM, RDA, and HFR reactions were detected (Figure 4B–G, Table 1). In addition, when one A-type linkage is present in procyanidins, the signals are shifted by -2 mass units from the signals of B-type procyanidins, corresponding to two hydrogen atoms that are lost during the formation of the extra interflavanic linkage (Figure 1B,C). Therefore, we assigned the peak at *m*/*z* 577.1 as B-type procyanidin dimers [M − H]^−^, while the peak at *m*/*z* 575.1 was assigned as A-type procyanidin dimers [M − H]^−^ with one A-type linkage [2,3,6,26] (Table 1). Similarly, other peaks were assigned as follows; *m*/*z* 861.2 as A-type procyanidin trimers with two A-type linkages; *m*/*z* 863.2 as A-type procyanidin trimers with one A-type linkage; *m*/*z* 1149.3 as A-type procyanidin tetramers with two A-type linkages; *m*/*z* 1151.3 as A-type procyanidin tetramers with one A-type linkage [2,3,6,26] (Table 1). From these results, it was speculated that that flavan-3-ols are biosynthesized in the outer epidermis of the peanut testa [1].

## 3. Discussion

In the present study, we used matrix vapor deposition/recrystallization as the matrix-coating method for visualizing procyanidins in MALDI-MSI. We found several procyanidin peaks by extraction of mass spectrum of the outer epidermis of the testa in the peanut sections. Using MALDI-MS/MS analysis of the outer epidermis of the testa in peanut sections, we assigned procyanidin monomers, (epi)catechin, five A-type and one B-type procyanidins, namely A-type procyanidin dimers with one A-type linkage, trimers with one and two A-type linkages, and tetramers with one and two type A-type linkages, and B-type procyanidin dimers (Table 1). The assigned procyanidins were visualized by MALDI–MSI analysis, which revealed the characteristic localization of procyanidins in peanut testa (Figure 3C–I).

In the MALDI-MS/MS analysis, the precursor ion selection window was set at a 1% *m*/*z* value for the precursor ion; however, the difference in mass between the A type and B type procyanidin dimers is only 2 Da (Figure 1B,C), indicating that each MS/MS spectrum contained composite fragment ions generated from each dimer (Figure 4B,C). Similar problems were encountered for the trimers (Figure 4D,E) and tetramers (Figure 4F,G), due to the present limitations of the LIFT MS/MS technique, suggesting that these MS/MS spectra were not specific to each procyanidin oligomer. The quadrupole (Q) mass filter is commonly more effective for the selection of precursor ions than LIFT. Therefore, a MALDI-MSI mass spectrometer equipped with Q may aid in overcoming these limitations.

Yu et al. [3] quantified flavan-3-ols, namely, procyanidin monomers, A-type and B-type procyanidin dimers, A-type and B-type procyanidin trimers, and A-type and B-type procyanidin tetramers in peanut skin extracts, via LC-MS and HPLC analysis. In the literature, several different molecular species for each flavan-3-ol species were reported to have been detected, based on the difference in the retention time, e.g., the detected number of peaks was six for A-type procyanidin trimers with one A-type linkage. This is commonly caused by the differences of the balance of catechin and epicatechin units or their positions and the position of the A-type linkage in the structure of each flavan-3-ol. Therefore, it is speculated that the flavan-3-ol species assigned in this study also contain several different molecular species (Table 1). Yu et al. also reported the detection of B-type procyanidin timers in peanut skin extracts, whereas the trimers were not detected in this study. In addition, in this study, A-type procyanidin trimers with two A-type linkages were detected, whereas such trimers were not reported in the literature [3]. The other flavan-3-ols assigned in this study were also reported in the literature [3]. This discrepancy might be due to the differences in the cultivar.

We showed that procyanidins were localized in the outer epidermis of the peanut testa (Figure 3C–I). The peanut skin is a valuable source of procyanidins, especially A-type procyanidins, which are extracted and further purified for use in the cosmetic or nutraceutical industries [2,27]. However, purification of procyanidins is commonly complicated due to their predominant contamination with other metabolites, such as lipids. Alternatively, the extraction of procyanidins exclusively from the outer epidermis of the peanut testa, instead of the entire hull, may make their purification more simple.

In angiosperms, flavan-3-ols help protect against ultraviolet radiation and ozone by reducing oxidative stress [1,2]. Tannins have been shown to be toxic to bacteria [1,2]. In addition, flavan-3-ol monomers and proanthocyanidins may also inhibit the germination of fungal spores and block the biosynthesis of melanin [1,2], which is an important factor in the virulence of many plant pathogenic fungi. In our previous study using strawberry fruit [20], flavan-3-ols were found mainly distributed in the calyx, in and around the vascular bundles, and in the skin. The distribution of flavan-3-ols in strawberry fruit was related to their protective function [12,20]. Pathogens such as bacteria and fungi infect plant surface tissues such as the testa. In nature, peanut seeds develop in the soil, where they are not reached by sunlight. Therefore, flavan-3-ols, especially A-type procyanidins, may be present in the outer epidermis of the peanut testa, to prevent the initial steps of pathogen invasion.

In our previous study using MADLI-MSI analysis of flavan-3-ols in strawberry fruit [20], we observed in-source fragmentation of procyanidin species by some reactions containing QM reaction. Therefore, ion images of assigned procyanidins showed in this study are mixed ion images of the ion itself and fragment ions generated from procyanidins with a larger degree of polymerization, because measurement conditions were similar to the previous study [20]. To visualize each flavan-3-ol individually using MSI, it may be necessary to develop a softer ionization mass-spectrometer than the vacuum MALDI-TOF/TOF used in this study. This also implies that the fragment ions observed in the MS/MS spectrum of each assigned flavan-3-ol species (Figure 4A–G, Table 1) were mixed fragment ions derived from not only an individual flavan-3-ol, but also from other flavan-3-ols with a larger degree of polymerization. In addition, Chen et al. [28] reported the conversion of B-type to A-type trimers by QM reaction mechanisms, implying that B-type procyanidins convert to A-type procyanidins by MALDI-MSI. Therefore, other analytical data, such as LC-MS/MS or NMR analyses are necessary when those data are not available as to ensure accurate molecular identification.

In conclusion, we demonstrated that DAN vapor deposition/recrystallization in MALDI-MSI is a useful tool for investigating the localization of flavan-3-ols in the peanut testa. This technique might be adaptable for investigating the localization of flavan-3-ols in other plant species and tissues. We expect that MALDI-MSI analysis and the spatial information of procyanidins in the peanut testa obtained in this study will further contribute to the fields of food and plant sciences.

## 4. Materials and Methods

### 4.1. Materials and Reagents

Methanol, water, and acetic acid were purchased from Wako Chemicals (Tokyo, Japan). DAN and α-cyano-4-hydroxycinnamic acid (CHCA) were purchased from Tokyo Kasei Co. (Tokyo, Japan). Indium-tin-oxide (ITO)-coated glass slides (100 Ω without MAS coating) were purchased from Matsunami Glass (Osaka, Japan). Peptide calibration standards containing angiotensin II, bombesin, and ACTH dip (1–17) were purchased from Bruker (Billerica, MA, USA). All reagents and solvents used in this study were of analytical grade.

### 4.2. Peanut (Arachis hypogaea L.) Samples

“Chiba-handachi” peanuts were cultivated in the farm managed by the Department of Biosciences at Teikyo University (Utsunomiya, Japan). Raw peanuts were harvested, frozen on the day of harvest, and stored at −80 °C until use.

### 4.3. Preparation of Peanut Sections

Peanut sections were prepared as described previously [13]. Briefly, longitudinal sections (50 μm thick) of frozen peanuts (600–1400 mg) were consecutively prepared using a CM 1860 cryostat (Leica Microsystems, Wetzlar, Germany). The sections were then mounted onto ITO-coated glass slides and placed in 50 mL conical centrifuge tubes containing silica gel for drying. The sections were preserved at −80 °C in a freezer until MALDI-MSI analysis.

### 4.4. Matrix Coating

Matrix coating was performed by matrix vapor deposition and subsequent matrix recrystallization. An ITO-coated glass slide with a frozen section was drawn from the freezer and dried in a vacuum desiccator for 30 min. Matrix vapor deposition was performed using the vacuum deposition system (SVC-700TMSG, Sanyu Electron Co., Ltd., Tokyo, Japan), according to Shimma et al. [25], with minor modifications. The glass slide was held to the sample holder using adhesive tape, and DAN (50 mg) was placed on the matrix holder. The distance between the sample holder and the matrix holder was maintained at 5 cm. After the vacuum pressure in the chamber reached to 5 × 10^3^ Pa, DAN was heated at 200 °C, until all the DAN powder sublimes.

Matrix recrystallization was performed according to Meisenbichler et al. [24] with some modifications. One mL of 5% aqueous methanol containing 10 mM acetic acid was placed in a glass Petri dish (90 mm in diameter), and preheated at 37 °C on the hot plate for 10 min. After matrix vapor deposition, the glass slide was kept at 37 °C in the closed Petri dish for 30 min, in order to expose the DAN on the section to the solvent vapors.

### 4.5. MALDI-MSI Analysis of Peanut Sections

MALDI-MSI analysis was performed, as in our previous study [20], after minor modifications. The peanut sections were analyzed using a MALDI-TOF/TOF instrument (ultrafleXtreme, Bruker, Billerica, MA, USA), equipped with a 355 nm Nd:YAG laser, using a repetition rate of 1000 Hz. Data were acquired using a 50-μm step size in negative-ion and reflector modes. The *m*/*z* values in the range 280–2000 were measured. The laser diameter was set to the medium size. The instrument was calibrated externally using the exact *m*/*z* values of CHCA [M − H]^−^ ions (*m*/*z* 188.03532), angiotensin II [M − H]^−^ ions (*m*/*z* 1044.52725), bombesin [M − H]^−^ ions (*m*/*z* 1617.80775), and ACTH dip (1–17) [M − H]^−^ ions (*m*/*z* 2091.07165) as references. The spectra were acquired automatically using flexImaging 4.1 software (Bruker, Billerica, MA, USA). Normalization of spectra based on the total ion current was performed using the same software, which was also used to create two-dimensional ion-density maps and extract mass spectrum from the outer epidermis of peanut testa using the region of interest function.

To investigate the spatial localization of the detected flavan-3-ols, three different peanuts were analyzed. The mass spectra and ion images of the detected flavan-3-ols in the three different peanuts showed similar patterns (Supplementary Figure S1). The mass spectrum and ion images of one of the three different peanut are presented as representative data in Figure 3.

### 4.6. MALDI-MS/MS Analysis of Peanut Sections

MALDI-MS/MS analysis was performed as in our previous study [20] after minor modifications. Briefly, the MS/MS spectra were obtained from the outer epidermis of testa in peanut sections after performing MALDI–MSI analysis using an ultrafleXtreme instrument operated in collision-induced dissociation “LIFT” MS/MS mode. The *m*/*z* values of precursor ions were set at each value ± 1%. The MS/MS spectra were analyzed using flexAnalysis 3.4 software (Bruker, Billerica, MA, USA). The molecular species of procyanidins detected in the peanut sections were assigned based on typical neutral losses observed after RDA, HRF, and QM reactions [2,26].

## Figures and Tables

**Table 1:** Assigned flavan-3-ol species in peanut testa.

Precursor Ion [M − H]^−^, (*m*/*z*)	Flavan-3-ol Species	Number of Linkage		Fragment Ions for Assignment [M − H]^−^, (*m*/*z*)		
		A-Type	B-Type			
289.1	Catechin and/or epicatechin			245, 125		
				QM	RDA	HRF
575.1	A-type procyanidin dimers	1	0	289, 285	407	449
577.1	B-type procyanidin dimers	0	1	289, 287	425, 407	451
861.2	A-type procyanidin trimers	2	0	575, 289, 285	693, 407	735, 449
863.2	A-type procyanidin trimers	1	1	575, 573, 289, 287, 285	711, 695, 693, 407	737, 451, 449
1149.3	A-type procyanidin tetramers	2	1	863, 861, 575, 573, 289, 287, 285	997, 981, 979, 711, 695, 693, 425, 407	1023, 737, 735, 451, 449
1151.3	A-type procyanidin tetramers	1	2	863, 861, 575, 573, 289, 287, 285	999, 981, 711, 695, 693, 425, 407	1025, 739, 737, 451, 449

Matrix-assisted laser desorption/ionization-tandem mass spectrometry (MALDI-MS/MS) spectra were obtained from the outer epidermis of the testa in peanut sections after MALDI-MS imaging analysis, using an ultrafleXtreme instrument operated in collision-induced dissociation “LIFT” MS/MS mode. Fragment ions obtained by quinone methide (QM), retro-Diels-Alder (RDA), and heterocyclic ring fission (HRF) reactions were used for molecular assignments [2,26].

## Supplementary Materials

The following are available online at https://www.mdpi.com/1420-3049/25/10/2373/s1. Figure S1: Representative ion images of the assigned flavan-3-ol species in peanut longitudinal sections by matrix-assisted laser desorption/ionization-mass spectrometry imaging. These ion images were obtained from two peanuts different from that shown in Figure 3. The dotted white line shows the analyzed region. Scale bar = 2 mm.
