# Supplementary material for: Localization of Flavan-3-ol Species in Peanut Testa by Mass Spectrometry Imaging
Source: Molecules. 2020 May 20;25(10):2373. doi: 10.3390/molecules25102373 (PMC7287834; doi:10.3390/molecules25102373)
Supplement: Supplementary file 1 [file molecules-25-02373-s001.pdf]

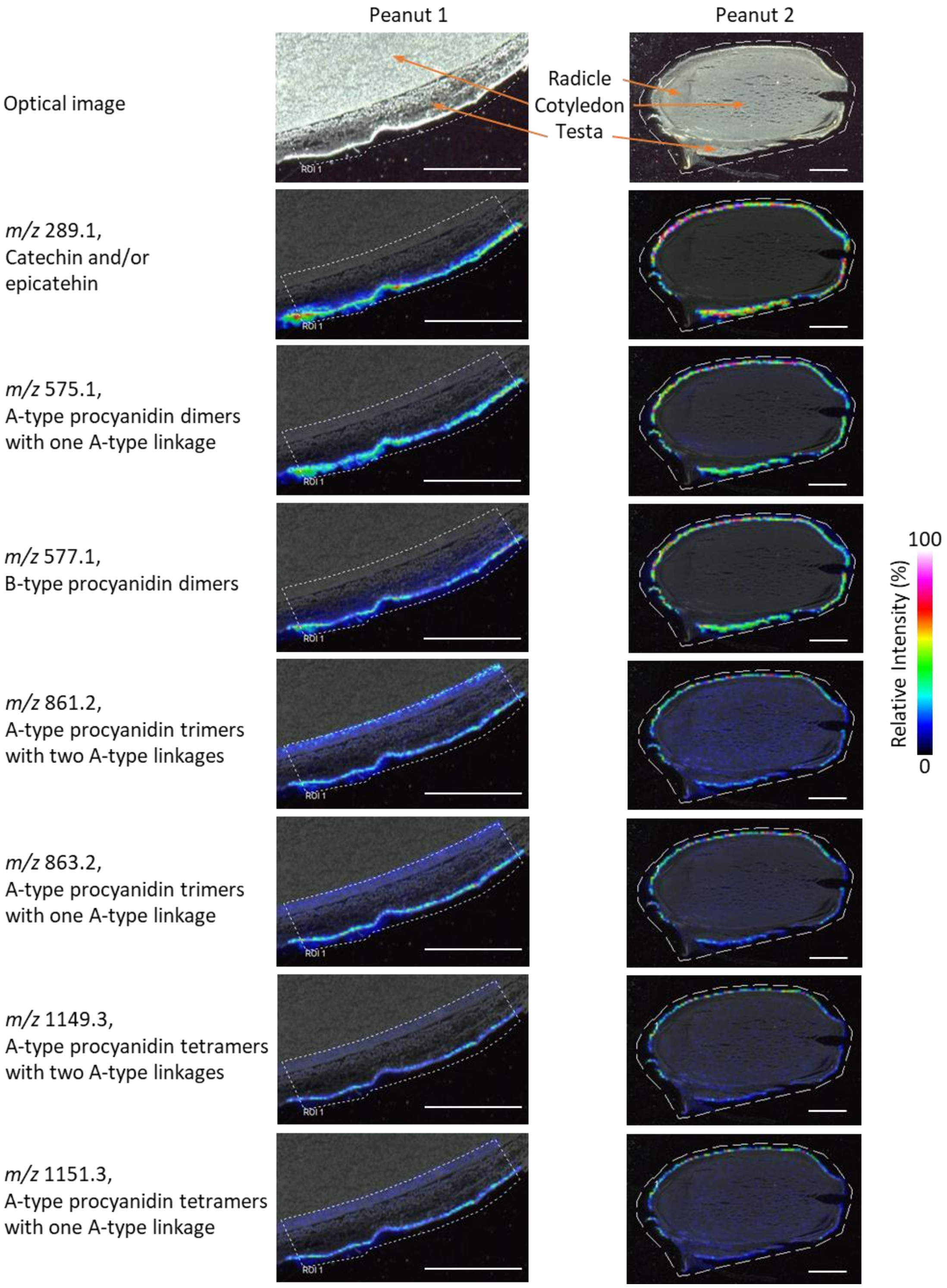

Supplementary Figure S1. Representative ion images of the assigned flavan-3-ol species in peanut sections by matrix-assisted laser desorption/ionization-mass spectrometry imaging. These ion images were obtained from two peanuts different from that shown in Figure 3. The dotted white line shows the analyzed region. Scale bar = 2 mm.
